# A Chromosome-Level Genome Assembly of the Dark Sleeper *Odontobutis potamophila*

**DOI:** 10.1093/gbe/evaa271

**Published:** 2021-02-12

**Authors:** Yongyi Jia, Jianbo Zheng, Shili Liu, Fei Li, Meili Chi, Shun Cheng, Zhimi Gu

**Affiliations:** Key Laboratory of Genetics and Breeding, Zhejiang Institute of Freshwater Fisheries, Huzhou, China

**Keywords:** *Odontobutis potamophila*, whole-genome sequence, sex determination, gene annotation

## Abstract

The dark sleeper, *Odontobutis potamophila*, is a commercially valuable fish that widely distributed in China and Southeast Asia countries. The phenomenon of sexual dimorphism in growth is conspicuous, which the males grow substantially larger and faster than the females. However, the high-quality genome resources for gaining insight into sex-determining mechanisms to develop sex-control breeding are still lacking. Here, a chromosomal-level genome assembly of *O. potamophila* was generated from a combination of Illumina reads, 10× Genomics sequencing, and Hi-C chromatin interaction sequencing. The assembled genome was 1,134.62 Mb with a contig N50 of 22.25 Mb and a scaffold N50 of 24.85 Mb, representing 94.4% completeness (Benchmarking Universal Single-Copy Orthologs). Using Hi-C data, 96.49% of the total contig bases were anchored to the 22 chromosomes, with a contig N50 of 22.25 Mb and a scaffold N50 of 47.68 Mb. Approximately 54.18% of the genome were identified as repetitive elements, and 23,923 protein-coding genes were annotated in the genome. The assembled genome can be used as a valuable resource for molecular breeding and functional studies of *O. potamophila* in the future.

**Significance**

A great many of omics (transcriptome, proteomics, and metabolomics) studies of *Odontobutis potamophila* have been reported in recent years. However, to solve the bottleneck problem in the breeding, the whole-genome sequencing of *O. potamophila* is necessary. Here, a chromosomal-level genome assembly was generated, which would allow for the study of many biological questions.

## Introduction

The dark sleeper *Odontobutis potamophila* is a commercially valuable fish that widely distributed in the river systems of China and Southeast Asia countries (Viet Nam, Japan, and Korean) (Hou et al. 2014; Zhang et al. 2015; Cheng et al. 2017). The aquaculture of this species is potential value due to their high meat content, delicious taste and high profits (Li and Liu 2016; Wang et al. 2017). As an unique economic fish from China, artificial breeding of *O. potamophila* was explored in early 1990s, but a significant breakthrough was made for large scale seedling in 2009. However, the current culture model of *O. potamophila* was mainly mixed with other aquatic species, resulting in the production were not enough to meet the increasing consumption demand (Yan-Dong et al. 2015).

Recently, a great deal of studies involved in *O. potamophila* have been carried out on reproduction, farming and larvae rearing (Liu et al. 2008; Zhao et al. 2009). Phylogeny analysis via mitochondrial 12S *rRNA* sequence demonstrated that Chinese odontobutis mainly consisted of four species, comprising *O. potamophila*, *Odontobutis sinensis*, *Odontobutis haifengensis*, and *Odontobutis yaluensis*, respectively. Many scholars also conducted some research on the toxicology experiments of *O. potamophila*, and found that chlorpyrifos could cause serious damage to the gill and liver in the larval stage (Ding et al. 2013). In addition, Zhang et al. (2014) developed many polymorphic microsatellite markers for the purpose of kinship identification, linkage map construction, and genetic diversity analysis. Furthermore, many omics studies had been performed in recent years to analyze the regulatory mechanism of relevant economic traits (Wang et al. 2019).

Unfortunately, seedlings showed the phenomenon of germplasm decay during the course of production, including slow growth rate, weak disease resistance, increased disease incidence. On the other hand, *O. potamophila* showed a sexually dimorphic growth pattern, which the males grew substantially larger and at a quicker rate than the females (Cheng et al. 2017). Therefore, elucidation growth- or sex-regulatory mechanism, and breeding with rapid growth merit of *O. potamophila* is of great significance for the genetic management and scientific research. To solve these bottleneck problems in the breeding of *O. potamophila* and clarify the biological characteristics at molecular level, it was imperative to initiate the whole-genome sequencing. Here, we reported the whole-genome sequence of *O. potamophila*, and the availability of reference genome will provide valuable resources for sex-control breeding and functional genomic research.

## Materials and Methods

### Sample Collection and Sequencing

The dark sleeper *O. potamophila* was obtained from the Balidian breeding base of Zhejiang Institute of Freshwater Fisheries in 2019 (Huzhou, China). The muscle tissues were dissected from a single female individual for DNA extraction using the phenol/chloroform extraction method. Library preparation and sequencing were performed by an external service (Novogene Co., Ltd., Beijing, China). High quality genomic DNA were randomly sheared (insert size 350 bp) through Covarisg-TUBE, and paired-end (PE) libraries were constructed for sequencing on the PromethION platform at Novogene (Beijing, China).To aid genome annotation, eight tissues from the same individual, including gill, heart, brain, muscle, intestine, skin, and ovary, were collected for RNA extraction and Transcriptome sequencing. Subsequently, sequencing libraries were prepared using NEBNextUltraTM RNA Library Prep Kit for Illumina (NEB) following manufacturer’s recommendations. Finally, the library preparations were sequenced on an Illumina platform and 125 bp/150 bp PE reads were generated.

### Genome Estimation and Assembly

For a general judgment of genome size, we used *K*-mer analysis to estimate genome size from the mathematical perspective. As a result, the genome size of *O. potamophila* was estimated to ∼1,156.17 Mb by the *K*-mer frequency distribution. The Illumina sequence reads were then assembled using Soapdenovo software as described below: 1) fragments randomly sheared into different insert sizes, 2) represent read sequence overlap using de Bruijin graph, 3) remove erroneous connections on the graph, 4) break at repeat boundaries and out contigs, 5) scaffold construction, and 6) gap closure (Li et al. 2010). Finally, the resulting assembly contigs were connected to linked-reads from 10× Genomics-derived sequencing data to yield a draft *O. potamophila* genome assembly (Adey et al. 2014).

To further improve the accuracy of the assembly, Hi-C libraries were constructed to generate a chromosome-level assembly of the genome. Hi-C clean data were mapped to the draft assembled sequence from 10× Genomics using BWA software (Servant et al. 2015), and the low quality reads were removed by SAMTOOLS. Last, the valid Hi-C reads pairs were applied for clustering, ordering, and orienting to finish aid assembly at a chromosome-level. Further, the completeness of the *O. potamophila* genome was evaluated by Core Eukaryotic Genes Mapping Approach (CEGMA) (Parra et al. 2007) and Benchmarking Universal Single-Copy Orthologs (BUSCO) (Simao et al. 2015), respectively.

### Repeat Analysis and Noncoding Gene Annotation

The repeat sequences in the genome mainly consisted of tandem repeat and interspersed repeat (Ge et al. 2019). Here, repetitive sequences annotation was performed by homology searches against known repeat databases and *de novo* prediction. Homology searches repetitive elements in the *O. potamophila* genome depended on the RepBase database (http://www.girinst.org/repbase/) with Repeatmasker and repeatproteinmask software (Bao et al. 2015). For the second method, de novo repetitive element database was firstly constructed by LTR_FINDER (Xu and Wang 2007), RepeatScout (Price et al. 2005), RepeatModeler (Smit and Hubley 2010) with default parameters, then employing the RepeatMasker (Tempel 2012) to annotate repeat elements with the database.

Noncoding RNAs, including miRNA, snRNA, tRNA, and rRNA, also have important biological functions (Hombach and Kretz 2016). For example, MicroRNAs (miRNAs) are small endogenous RNAs that regulate gene-expression posttranscriptionally in many different cellular pathways and systems (Lu and Rothenberg 2018). Transfer RNAs (tRNAs) and Ribosomal RNAs (rRNAs) are thought to directly involve in protein synthesis (Jarroux et al. 2017). Small nuclear RNA (snRNA) is proven to participate in pre-mRNA splicing (Shi 2017). These noncoding RNAs were identified and annotated across the *O. potamophila* genome. The tRNAs were predicted using the program tRNAscan-SE (Lowe and Chan 2016). High conserved rRNAs were annotated using BlastN (Camacho et al. 2009), and other ncRNAs were identified by searching against the Rfam database with default parameters using the infernal software (Daub et al. 2015).

### Protein-Coding Gene Prediction and Annotation

Gene models were established using a combination of ab initio, homology-based and RNA-Seq assisted prediction. For gene predication based on ab initio, we employed Augustus (v3.2.3), Geneid (v1.4), Genescan (v1.0), GlimmerHMM (v3.04), and SNAP (2013-11-29) to predict protein-coding genes in *O. potamophila* genome (Korf 2004; Majoros et al. 2004; Stanke et al. 2004). Regarding homology-based prediction, proteins sequences of *Gadus morhua*, *Ctenopharyngodon idellus*, *Cyprinus carpio*, and *Larimichthys crocea*, were downloaded from Ensembl/NCBI/others. Subsequently, potential gene structures were aligned to the homologous genome for all alignments with GenWise software (v2.4.1) (Birney and Durbin 2000). For transcriptome-based prediction, RNA-seq data from different tissues were generated with Trinity (v2.1.1) for the genome, and exons region and splice positions were aligned to genome fasta using Hisat (v2.0.4)/TopHat (v2.0.11) with default parameters (Trapnell et al. 2012; Kim et al. 2013). Functional annotation of the predicted genes was performed using public databases of SwissProt, InterPro, NR from NCBI and Kyoto Encyclopedia of Genes and Genomes (KEGG). The motifs and domains were annotated using InterProScan by searching against protein databases, such as ProDom, PRINTS, Pfam, SMART, PANTER, and PROSITE.

### Comparative Genome Analysis

Gene families were analyzed using OthoMCL for identification species-specific and shared genes between *O. potamophila* and other ten fish species (Feng et al. 2006), including *L. crocea*, *Danio rerio*, *Gasterosteus aculeatus*, *Oreochromis niloticus*, *Takifugu rubripes*, *C. carpio*, *Cynoglossus semilaevis*, *C. idellus*, *Oncorhynchus mykiss*, and *Oryzias latipes.* To examine *O. potamophila* evolution, single-copy genes from the above analysis were selected for multi-alignment using MUSCLE (Robert 2004) to build super alignment matrix, and then a phylogenetic tree was constructed by RAxML software with ML TREE method. Subsequently, divergence time was estimated using PAML software (Yang 2007).

## Results and Discussion

### Genome Assembly and Statistics

**Fig. 1:**
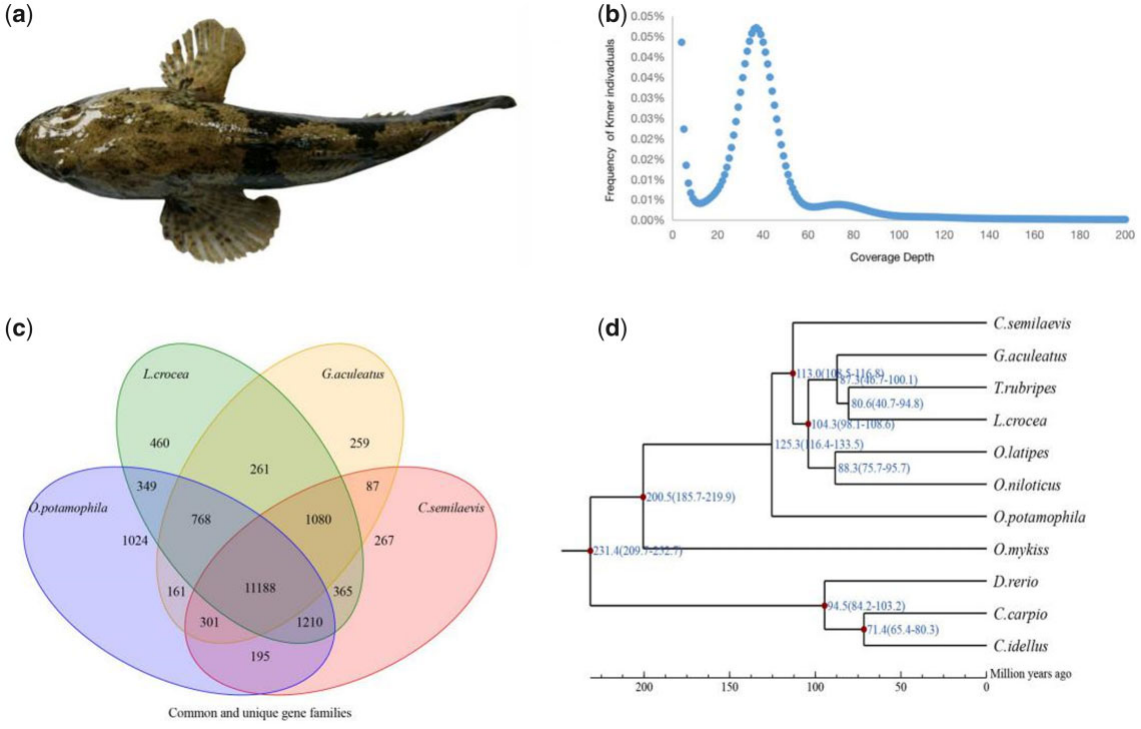
Assemblies and evolution of *Odontobutis potamophila* genome. (*a*) An adult dark sleeper. (*b*) 41-mer frequency distribution in the genomes. The *X*-axis is the Kmer depth, and *Y*-axis represents the frequency of the Kmer for a given depth. (*c*) Venn diagram of shared and unique orthologous gene family for four selected vertebrate genomes. Each number represents the number of orthologous gene families shared by the indicated genomes. (*d*) Phylogenetic relationship of *O. potamophila* and 10 other fish species genome using 1,182 single copy orthologous genes. The divergence time is given in millions of years in blue color. The relative rates of molecular evolution are expressed as the branch lengths. Estimates of divergence times (millions of years) calculated from the rate of sequence similarity are indicated at each node.

Here, we performed the whole-genome sequencing of *O. potamophila* with Oxford Nanopore technology on PromethION platforms. To estimate the genome size and heterozygosity of *O. potamophila*, 17-mers were counted as 42,778,163,910 from clean reads, and the size of genome was approximately 1,156.17 Mb with 0.29% heterozygosity by survey analysis (supplementary tables S1 and S2, Supplementary Material online, fig. 1). The detailed genome sequencing information was summarized, and a total of 181.27 G (coverage of 160.41×) clean data were produced after quality filtration from a single genomic DNA library (table 1). Accordingly, a final 1,134.62 Mb draft genome assembly was obtained, covering 98.14% of the estimated genome sizes. The N50s of contigs and scaffolds of the *O. potamophila* genome were 22.25 Mb and 24.85 Mb, respectively. The GC content of the assembly genome was estimated to be 43.26% (supplementary table S3, Supplementary Material online).

**Table 1:** Statistics of the Genome Sequencing Data

Pair-end Libraries	Insert Size	Total Data (G)	Read Length (bp)	Sequence Coverage (×)
Nanopore	—	130.43	—	115.42
Illumina reads	350	50.84	150	44.99
10×	600	121.55	—	107.57
Hi-C	—	113.07	—	100.06
Total	—	415.89	—	368.04

To improve the genome sequencing read-level accuracy, we used a combination of linked-reads and proximity ligation in this study. Using Hi-C data, 96.49% (1,058,372,153) of the total contig bases (1,096,900,524) were anchored to the 22 chromosomes, with a contig N50 of 22.25 Mb and a scaffold N50 of 47.68 Mb (supplementary table S1 and supplementary fig. S1, Supplementary Material online). Furthermore, the completeness of the *O. potamophila* genome was evaluated by CEGMA and BUSCO, respectively. Using CEGMA method, 95.56% of the 248 core genes were identified in the genome (supplementary table S4, Supplementary Material online), and BUSCO results revealed that 94.4% complete and 1.2% partial of the 2586 vertebrate BUSCO genes were captured (supplementary table S5, Supplementary Material online). Taken together, our results indicated that the genome assembly was complete and of high quality.

### Repeat Analysis and Genome Annotation

The identification of repetitive elements showed that a total of 587,699,328 bp repeat sequences were identified in the *O. potamophila* genome, which accounted for 53.58% of the genome. Among them, 0.49% of the genome was identified as tandem repeat, and long terminal repeat retrotransposons (48.75%) were the most abundant TEs in *O. potamophila*, followed by long interspersed elements (LINEs, 15.49%) (supplementary table S6, Supplementary Material online). The gene model prediction method was applied to the protein-coding gene annotation in the *O. potamophila* genome. For genome annotation, approximately 24,748 protein-coding genes were identified, and a total of 23,923 genes (96.7%) were annotated by at least one public database (supplementary table S7, Supplementary Material online). Furthermore, four types of noncoding RNAs were predicted across the *O. potamophila* genome, comprising 1,876 miRNAs, 1,569 rRNA, 4,139 tRNAs, and 654 snRNAs (supplementary table S8, Supplementary Material online).

### Comparative Genome Analysis

To investigate the phylogenetic position of *O. potamophila* with other published fish species, OrthoMCL was used for orthologue group identification. Clustering analysis revealed that 7,974 gene families and 1,182 single-copy genes were shared by *O. potamophila* and other fish species. Moreover, a total of 1,024, 460, 259, and 267 gene families were found specific to *O. potamophila*, *L. crocea*, *G. aculeatus*, and *C. semilaevis*, respectively (fig. 1). Using these single-copy orthologues, we constructed a phylogenetic tree by RAxML software with ML TREE method. Phylogenetic analysis showed that Cyprinidae family (*D. rerio*, *C. idellus* and *C. carpio*) clustered one branch, and *O. potamophila* was closely related to *C. semilaevis*, and the estimated divergence time was approximately 125 Ma (fig. 1).

## Conclusions

In the present study, we represented the chromosome-level genome sequencing, assembly, and annotation of *O. potamophila* using multiple sequencing platforms. The draft genome assembly was 1,134.62 Mb with a contig N50 of 22.25 Mb and a scaffold N50 of 24.85 Mb. The genome was functionally annotated to generate 24,748 protein-coding genes. The availability of the high-quality reference genome resource will be valuable for functional studies, especially elucidating on sex-determining mechanisms.

## Supplementary Material

Supplementary data are available at *Genome Biology and Evolution* online.

## Supplementary Material

evaa271_Supplementary_Data
