# Supplementary material for: A Chromosome-Level Genome Assembly of the Dark Sleeper Odontobutis potamophila
Source: Genome Biol Evol. 2021 Feb 12;13(2):evaa271. doi: 10.1093/gbe/evaa271 (PMC7883661; doi:10.1093/gbe/evaa271)
Supplement: evaa271_Supplementary_Data [file evaa271_supplementary_data.docx]

**Table S1 Summary of clean reads generated from genome and transcriptome sequencing**

| **Library ID** | **Sample** | **Raw Reads** | **Clean Reads** | **Clean Bases** | **Q20** | **Q30** | **GC content** |
| --- | --- | --- | --- | --- | --- | --- | --- |
| FRAS190032745-1a | Heart | 43,028,252 | 42,395,092 | 6.36G | 97.22 | 92.34 | 48.55 |
| FRAS190032746-1a | Brain | 57,597,628 | 56,759,028 | 8.51G | 97.40 | 92.71 | 47.76 |
| FRAS190032747-1a | Liver | 42,343,744 | 41,832,676 | 6.27G | 97.55 | 93.02 | 49.63 |
| FRAS190032748-1a | Muscle | 57,585,350 | 56,595,076 | 8.49G | 97.24 | 92.47 | 47.44 |
| FRAS190032749-1a | Gill | 49,553,110 | 49,129,540 | 7.37G | 97.35 | 92.64 | 48.58 |
| FRAS190032750-1a | Ovary | 46,116,652 | 45,544,230 | 6.83G | 96.91 | 91.99 | 49.54 |
| FRAS190032751-1a | Intestines | 49,669,006 | 49,142,036 | 7.37G | 97.45 | 92.86 | 50.61 |
| FRAS190032752-1a | Skin | 54,757,646 | 53,668,844 | 8.05G | 97.49 | 92.98 | 49.13 |
| FRAS190036645-1a | Hi-C_1 | 47,767,720 | 47,186,388 | 7.08G | 97.48 | 92.93 | 51.16 |
| FRAS190036646-1a | Hi-C_2 | 54,387,888 | 53,401,028 | 8.01G | 97.59 | 93.16 | 51.20 |

**Table S2 Estimation of genome size based on 17-mer statistics**

| **Kmer** | **Depth** | **N-kmer** | **Genome size (M)** | **Heterozygous rate (%)** |
| --- | --- | --- | --- | --- |
| 17 | 37 | 42,778,163.910 | 1156.17 | 0.29 |

**Table S3 Summary of the *O. potamophila* genome assembly**

| **Sample ID** | **length** | | **number** | |
| --- | --- | --- | --- | --- |
|  | Contig**(bp) | Scaffold**(bp) | Contig** | Scaffold** |
| Total | 1,096,393,887 | 1,096,900,524 | 1,461 | 1,281 |
| Max | 51,806,499 | 63,513,044 | - | - |
| Number>=2000 | - | - | 1,453 | 1,273 |
| N50 | 22,247,283 | 47,682,490 | 17 | 11 |
| N60 | 13,829,738 | 45,650,539 | 23 | 13 |
| N70 | 8,154,017 | 45,193,068 | 34 | 16 |
| N80 | 4,637,599 | 44,843,241 | 51 | 18 |
| N90 | 2,358,164 | 39,987,806 | 83 | 21 |

**Table S4 CEGMA assessment of the *O. potamophila* genome assembly**

| **species** | **complete** | | **complete + partial** | |
| --- | --- | --- | --- | --- |
|  | # Prots | %completeness | # Prots | %completeness |
| *O. potamophila* | 221 | 89.11 | 237 | 95.56 |

Complete：core gene with a completeness>70%；

Complete + partial：Complete and partial core gene；

#Prots：Number of core gene；

%completeness：the proportion core gene in the reference core gene library.

**Table S5 BUSCO assessment of the *O. potamophila* genome assembly**

| **Species** | **BUSCO notation assessment results** |
| --- | --- |
| *O. potamophila* | C:95.6%[S:94.4%,D:1.2%],F:3.4%,M:1.0%,n:2586 |

C：Complete BUSCOs

S：Complete and single-copy BUSCOs

D：Complete Duplicated BUSCOs

F：Fragmented BUSCOs

M：Missing BUSCOs

n：Total BUSCO groups searched

**Table S6 Summary statistics of annotated repeats**

| **Type** | **Denovo+Repbase** | | **TE proteins** | | **Combined TEs** | |
| --- | --- | --- | --- | --- | --- | --- |
|  | **Length(bp)** | **% in Genome** | **Length(bp)** | **% in Genome** | **Length(bp)** | **% in Genome** |
| DNA | 2,596,563 | 0.24 | 3,074,004 | 0.28 | 5,402,097 | 0.49 |
| LINE | 26,603,730 | 2.43 | 164,089,195 | 14.96 | 169,870,741 | 15.49 |
| SINE | 496,535 | 0.05 | 0 | 0 | 496,535 | 0.05 |
| LTR | 529,881,543 | 48.31 | 75,297,921 | 6.86 | 534,685,271 | 48.75 |
| Unknown | 14,246,690 | 1.30 | 0 | 0 | 14,246,690 | 1.30 |
| Total | 569,499,994 | 51.92 | 242,350,763 | 22.09 | 587,699,328 | 53.58 |

**Table S7 Summary statistics of functional annotated protein-coding genes**

|  | **Number** | **Percent(%)** |
| --- | --- | --- |
| Total | 24,748 | - |
| Swissprot | 21,165 | 85.50 |
| Nr | 23,886 | 96.50 |
| KEGG | 21,481 | 86.80 |
| InterPro | 22,335 | 90.20 |
| GO | 15,633 | 63.20 |
| Pfam | 19,703 | 79.60 |
| Annotated | 23,923 | 96.70 |
| Unannotated | 825 | 3.30 |

**Table S8 Summary statistics of non-coding RNA**

| **Type** | | **Copy number** | **Average length(bp)** | **Total length(bp)** | **% of genome** |
| --- | --- | --- | --- | --- | --- |
| miRNA | | 1,876 | 95.00 | 178,215 | 0.016247 |
| tRNA | | 4,139 | 74.33 | 307,663 | 0.028049 |
| rRNA | rRNA | 1,569 | 228.93 | 359,189 | 0.032746 |
|  | 18S | 247 | 365.10 | 90,180 | 0.008221 |
|  | 28S | 1,075 | 227.67 | 244,748 | 0.022313 |
|  | 5.8S | 56 | 120.46 | 6,746 | 0.000615 |
|  | 5S | 191 | 91.70 | 17,515 | 0.001597 |
| snRNA | snRNA | 654 | 144.79 | 94,693 | 0.008633 |
|  | CD-box | 126 | 118.40 | 14,919 | 0.001360 |
|  | HACA-box | 81 | 146.72 | 11,884 | 0.001083 |
|  | splicing | 429 | 149.39 | 64,089 | 0.005843 |
|  | scaRNA | 18 | 211.17 | 3,801 | 0.000347 |


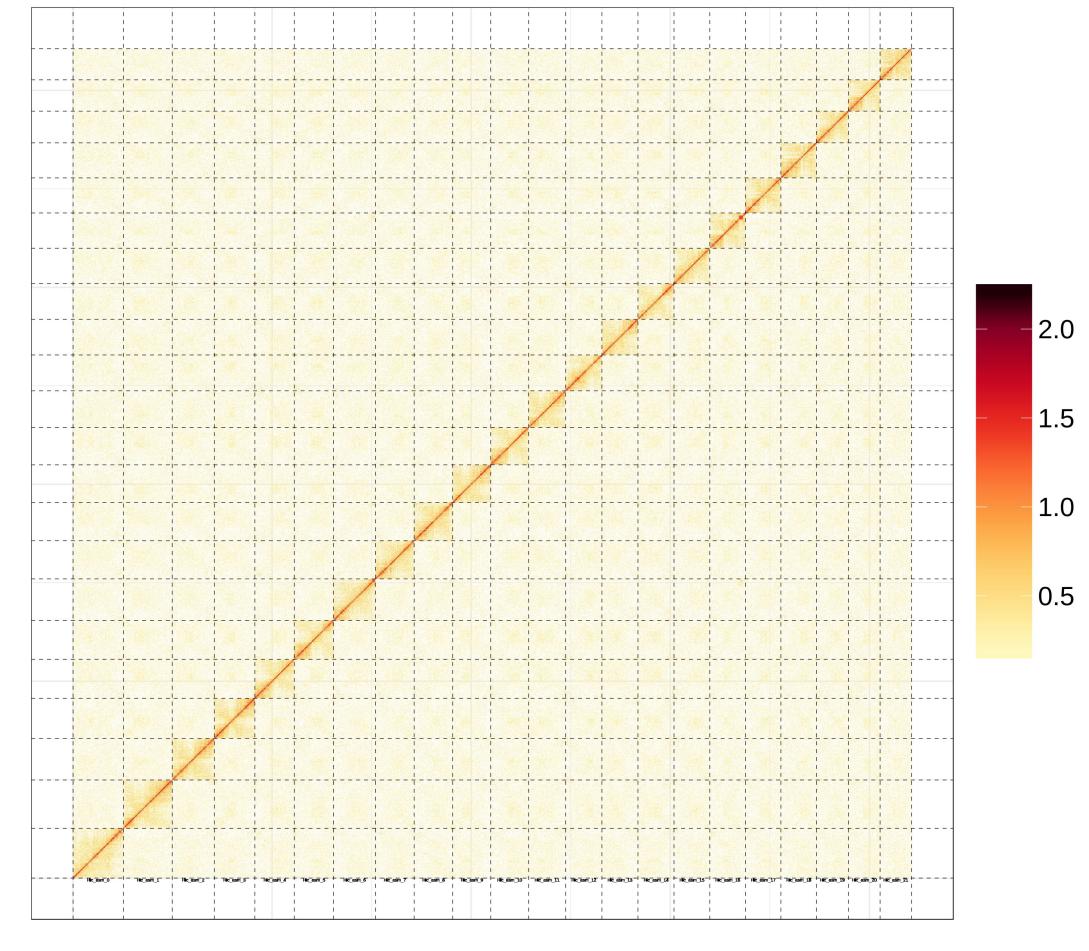


**Fig S1. Red-spotted grouper genome contig contact matrix using Hi-C data.**
